# Digital Dental Biometrics for Human Identification Based on Automated 3D Point Cloud Feature Extraction and Registration

**DOI:** 10.3390/bioengineering11090873

**Published:** 2024-08-28

**Authors:** Yu Zhou, Li Yuan, Yanfeng Li, Jiannan Yu

**Affiliations:** 1 School of Automation and Electrical Engineering, University of Science and Technology Beijing, 30 Xueyuan Road, Haidian District, Beijing 100083, China; m202220772@xs.ustb.edu.cn; 2 Key Laboratory of Knowledge Automation for Industrial Processes, Ministry of Education, 30 Xueyuan Road, Haidian District, Beijing 100083, China; 3 Department of Stomatology, the Fourth Medical Center, Chinese PLA General Hospital, 51 Fucheng Road, Haidian District, Beijing 100048, China; lyf304@301hospital.com.cn (Y.L.); yujiannan@301hospital.com.cn (J.Y.); 4 Department of Stomatology, the Sixth Medical Center, Chinese PLA General Hospital, 6 Fucheng Road, Haidian District, Beijing 100048, China; 5 Chinese PLA Medical School, 28 Fuxing Road, Haidian District, Beijing100853, China

**Keywords:** dental biometrics, 3D dental point cloud, human identification, coarse-to-fine registration, machine learning

## Abstract

Background: Intraoral scans (IOS) provide precise 3D data of dental crowns and gingival structures. This paper explores an application of IOS in human identification. Methods: We propose a dental biometrics framework for human identification using 3D dental point clouds based on machine learning-related algorithms, encompassing three stages: data preprocessing, feature extraction, and registration-based identification. In the data preprocessing stage, we use the curvature principle to extract distinguishable tooth crown contours from the original point clouds as the holistic feature identification samples. Based on these samples, we construct four types of local feature identification samples to evaluate identification performance with severe teeth loss. In the feature extraction stage, we conduct voxel downsampling, then extract the geometric and structural features of the point cloud. In the registration-based identification stage, we construct a coarse-to-fine registration scheme in order to realize the identification task. Results: Experimental results on a dataset of 160 individuals demonstrate that our method achieves a Rank-1 recognition rate of 100% using complete tooth crown contours samples. Utilizing the remaining four types of local feature samples yields a Rank-1 recognition rate exceeding 96.05%. Conclusions: The proposed framework proves effective for human identification, maintaining high identification performance even in extreme cases of partial tooth loss.

## 1. Introduction

Forensic dentistry is an important branch of forensic science that primarily utilizes the characteristics of teeth and jawbones for human identification, age estimation, and more [1]. The technology of using dental biometrics for human identification has significant applications in various fields. For instance, in forensic identification, when encountering mass disasters such as plane crashes, fires, explosions, etc., or serious crime scenes, frequently used identification technologies such as fingerprinting identification [2], DNA analysis [3], and facial identification [4] may be ineffective due to the destruction of related features. However, human tooth, as the hardest and most structurally stable tissue in the body, can often be well preserved in these extreme environments. Therefore, using dental biometrics for human identification become a reliable means of human identification in such scenarios [5]. In healthcare, a patient’s dental records contain detailed information about their treatment history. Using dental biometrics for human identification can assist doctors in confirming the patient’s identity, allowing them to access the patient’s dental records and make a more accurate diagnosis. In legal processes, the results of human identification using dental biometrics can be used as evidence to help resolve identity disputes in legal cases.

In the open literature, human identification based on dental biometrics can be categorized into three types according to the different data sources used: methods based on panoramic dental X-ray images, methods based on digitized dental plaster models, and methods based on data from Intraoral scans (IOS).

Chen et al. [6] integrated tooth contour characteristics with dental work characteristics (e.g., crowns, fillings, and bridges) to match teeth in dental X-ray images. Permata [7] proposed employing the active shape model to segment dental images in panoramic dental X-ray images in order to automatically fit the tooth contours. Subsequently, moment invariants were extracted as image features and the Euclidean distance between the moment invariants of two images was computed to gauge the image similarity, achieving image matching. Gurses et al. [8] employed Mask R-CNN for the segmentation and digital labeling of teeth within panoramic dental X-ray images. Subsequently, they extracted speeded-up robust features key points for each tooth and utilized them for matching purposes. The selection of the image demonstrating the highest overall similarity constituted the matching result. The methodology was validated through the matching of 102 images sourced from 51 unique individuals captured at different time points, resulting in a Rank-1 accuracy of 80.39% and Rank-7 accuracy of 100%. Fan et al. [9] proposed the DENT-net network aimed at human identification via panoramic dental X-ray images. Through experimentation on a test set comprising 326 such images, the network demonstrated high performance, with an accuracy rate of 85.16% at Rank-1 and 97.74% at Rank-5. Lai et al. [10] introduced LCA-Net, a novel approach designed to facilitate the matching of panoramic dental X-ray images for human identification purposes. LCA-Net incorporates a connected module featuring a learnable weighting scheme alongside an enhanced channel attention module to augment the network’s identification capabilities. Evaluation conducted on a test dataset comprising 1168 images revealed promising results, with an accuracy rate of 87.21% at Rank-1 and 95.34% at Rank-5. Zhong et al. [11] proposed a pose-invariant 3D dental biometrics framework including a multi-scale feature extraction algorithm for extracting pose invariant feature points and a triplet-correspondence algorithm for pose estimation, with digitized dental plaster models used as experimental data. The combination of the two algorithms in this framework can quickly and accurately perform feature point extraction and correspondence. Zhong et al. [12] attempted to automatically extract dental arches and to perform tooth matching and identification. They proposed a radial ray algorithm to project the dental arch shape of a digitized dental plaster model from 3D to 2D, and subsequently employed the extracted 2D dental arch characteristics for human identification through matching.

The aforementioned approaches predominantly rely on either panoramic dental X-ray images or digitized dental plaster samples for identification purposes. However, the utilization of panoramic dental X-ray images for identification involves projection of the three-dimensional dental structure onto a two-dimensional plane image. This process results in the expansion of the model from its radian structure into a planar representation, leading to a loss of crucial spatial structural information. Notably, spatial structural features play a pivotal role in the task of human identification using tooth. Moreover, the process of acquiring digitized dental plaster models is cumbersome, characterized by low sampling efficiency and poor comfort levels during sampling. Consequently, this model finds limited utility in clinical applications.

In recent years, the advancement of intraoral scanning technology has sparked increased interest in human identification utilizing IOS data. Reesu et al. [13] proposed superimposing two 3D models and calculating the alignment degree to realize human identification; however, a drawback is that the data contain both crown and gingival information after preprocessing, which results in a large number of points that are irrelevant to identification. Gibelli et al. [14] utilized a 3D–3D superimposition technique to analyze morphological disparities between the maxillary first and second molars in digital models. Nonetheless, their focus remained limited to inter-individual differences in these specific molars. Mou et al. [15] proposed performing 3D superimposition on the two 3D point clouds to be identified, and applied a correntropy-based ICP algorithm to calculate the superimposed RMS values in order to determine the similarity of the two point clouds, thereby achieving the identification function. Although the data involved in superimposed registration only include crowns, the amount of data is still large and not all points are useful for identification. Notably, the absence of preregistration prior to employing the iterative closest point (ICP) algorithm hinders its registration performance. Additionally, this study only focused on upper dentition.

The present work aims to address the challenges encountered in prior works [13,14,15], focusing on using 3D dental point cloud data provided by IOS to achieve human identification. To address the aforementioned issues, we propose a dental biometrics framework for human identification. To prevent the participation of a large number of irrelevant points in the identification process, the proposed framework extracts key points that represent the unique geometric features of tooth from the IOS of the maxillary and mandibular regions as holistic feature identification samples. This approach can effectively filter out irrelevant points. To enhance registration performance, the proposed framework utilizes machine learning-related algorithms to perform feature extraction and coarse-to-fine registration. Additionally, the framework constructs four types of local feature identification samples based on the holistic feature identification samples in order to evaluate the proposed method’s performance in scenarios involving partial tooth loss. Through our proposed framework and corresponding experimental outcomes, we aim to furnish valuable insights for researchers utilizing IOS for identification purposes.

## 2. Materials and Methods

### 2.1. Sample Description

This study recruited 160 adults aged from 18 to 48 who were receiving dental treatment. The study sample included 59 males and 101 females. All patients are from the Han population in the area of Beijing, China. The maxillary and mandibular dentition were all complete, with no severe crowding, no missing teeth, no supernumerary teeth, no deformed teeth, and no periodontal disease. The exclusion criteria covered patients who were unable to cooperate due to disease, had received or were undergoing orthodontic treatment, had undergone orthognathic surgery, or had either congenital edentulism or missing dentition.

**Figure 1:**
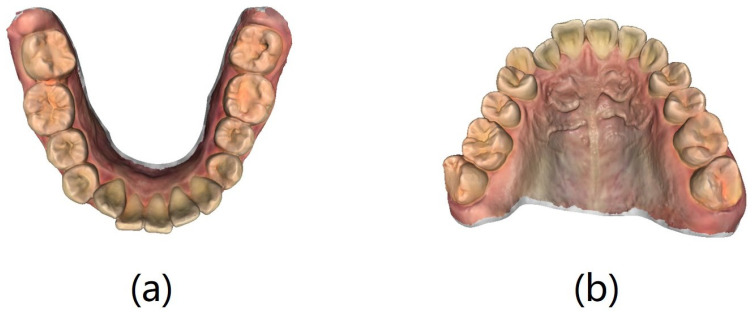
Intraoral scan data: (**a**) mandibular model and (**b**) maxillary model.

IOS models of all patients were acquired by the same trained oral surgeon using an iTero Element 3D intraoral scanner (Align Technology Ltd., San Jose, CA, USA), as shown in Figure 1. The data were exported to the Polygon file format (.ply) for subsequent use. Two sets of IOS data were created, the first including all 160 patients (gallery dataset) and the second (query set) including 72 of the 160 patients after one year.

### 2.2. Method Overview

**Figure 2:**
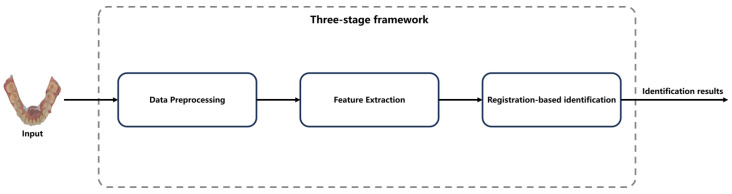
Proposed three-stage framework of dental biometrics for human identification.

The proposed dental biometrics framework consists of the three stages shown in Figure 2. The data preprocessing stage aims to extract key points that retain the geometric features of the tooth from IOS data, serving as samples for subsequent human identification. The feature extraction stage focuses on deriving feature information from the samples, which can facilitate point cloud registration. This extraction process can be achieved through the machine learning-related algorithms, as proposed in this paper, or through deep neural networks such as PointNet [16]. During the registration-based identification stage, the query point cloud is registered one-by-one with each point cloud in the gallery dataset. This registration process can be implemented using the machine learning-related algorithms proposed in this paper, or through deep neural networks such as CoFiNet [17]. The identity information of the query point cloud is determined based on the final registration results. This paper focuses on an exploratory study of 3D dental point clouds used for human identification. Considering the size of the dataset, machine learning-related algorithms are employed to handle tasks in both the feature extraction and identification stages.

**Figure 3:**
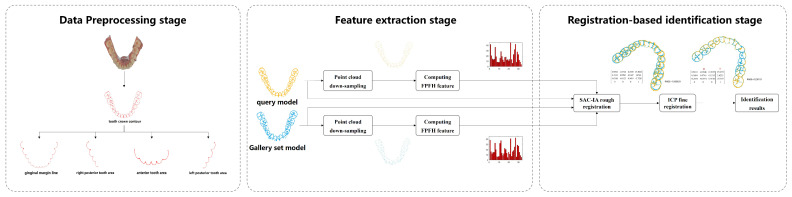
Detailed system diagram of human identification based on the proposed three−stage framework.

Specifically, in the data preprocessing stage we utilize the curvature principle to extract the tooth crown contour from IOS data. This serves as the holistic feature identification sample. We further derive four types of local feature identification samples based on these tooth crown contour samples. During the feature extraction stage, we combine the voxel downsampling method with the fast point feature histograms (FPFH) descriptor to capture the geometric and structural features of the identification samples, aiding in the subsequent identification process. Finally, in the registration-based identification stage, we integrate the sample consensus initial alignment (SAC-IA) coarse registration algorithm with the iterative closest point (ICP) fine registration algorithm, forming a coarse-to-fine registration process that achieves precise human identification. The specific details of the proposed framework are shown in Figure 3. To facilitate reader comprehension, Figure 3 illustrates the feature extraction and identification process using only the tooth crown contour sample as the identification sample. To evaluate the performance of the proposed method under extreme conditions of partial tooth loss, four types of local feature identification samples are individually used as identification samples. These samples undergo the same identification processing as the tooth crown contour.

### 2.3. Data Preprocessing Stage

In the data preprocessing stage, two types of identification samples are extracted from IOS data, namely, holistic feature identification samples and local feature identification samples. The local feature identification samples include four different types of features.

#### 2.3.1. Holistic Feature Identification Sample Extraction with Curvature Thresholding

**Figure 4:**
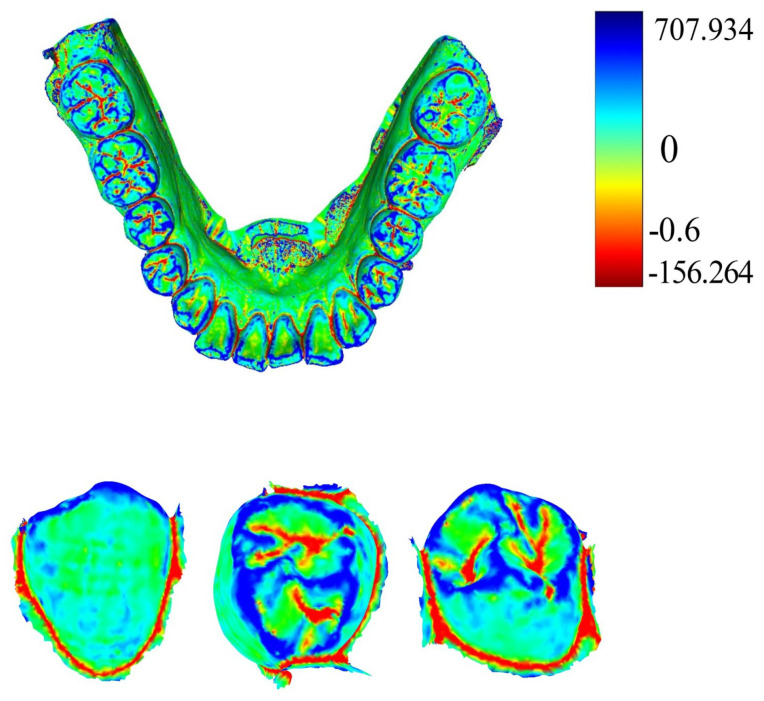
Curvature calculation visualization. The first row displays curvature results for IOS data, where the color bar indicates the correspondence between value and color. The second row focuses on curvature visualization for a single tooth.

Curvature serves as a crucial metric for quantifying the degree of bending in geometric objects such as curves and surfaces. Given a point on the tooth surface, acquiring its normal vector enables the generation of an infinite cutting plane centered around this vector. Each cutting plane interacts with the tooth surface to form a two-dimensional curve. Within this collection of curves, principal curvatures emerge that representing the maximum and minimum curvature values along the surfaces. The mean curvature, computed as the arithmetic mean of the principal curvatures, serves to gauge the convexity and concavity in IOS data. Utilizing the pseudo-inverse quadratic fitting technique, the mean curvature for each point on the IOS is computed. Figure 4 illustrates the visualization of these curvature calculations. Notably, the curvature values along the corresponding positions of the tooth crown contour consistently register below −0.6, as highlighted by the red dot in Figure 4. It is our belief that not all points within the point cloud contribute to human identification. Given the substantial variations in the geometric attributes of individual tooth crown contours, we propose that extracting and utilizing only the contour points can be effective for human identification purposes.

**Figure 5:**
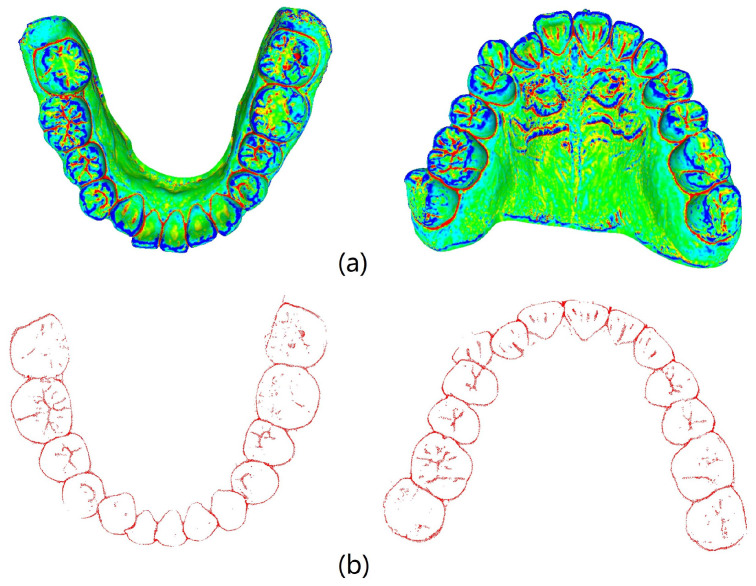
Mean curvature visualization and extraction of key points: (**a**) visualization results of mean curvature calculation for mandibular and maxillary models; (**b**) curvature feature extraction outcomes.

MeshLab v2020.09 (Visual Computing Lab, ISTI−CNR, Pisa, Italy) combined with the pseudo−inverse quadratic fitting method was utilized to calculate and color−code the average curvature of each point on the tooth model. Points with curvature values below −0.6 were extracted, and noise below the tooth outline was removed. Figure 5 illustrates the visualization of average curvature calculations and the tooth crown contour extraction results. The extraction of the tooth crown contours is evident, demonstrating an effective extraction process. We define the tooth crown contour feature as the holistic feature.

#### 2.3.2. Local Feature Identification Samples Extraction

**Figure 6:**
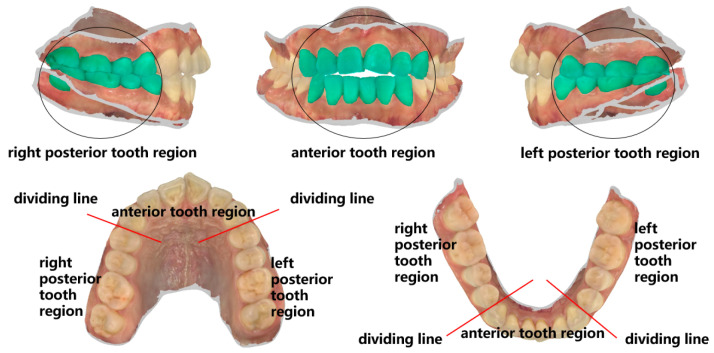
Schematic diagram of gingival margin cutting.

**Figure 7:**
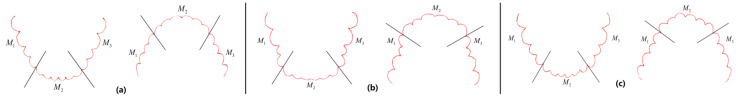
Images (**a**–**c**) respectively represent the details of the mandibular and maxillary gingival margins and subregional divisions in three cases; $M_{1}$ represents the right posterior tooth region, $M_{2}$ represents the anterior tooth region, and $M_{3}$ represents the left posterior tooth region.

To investigate missing features under extreme conditions, the feature points were selectively reduced based on the tooth crown contour features in order to simulate their absence. Building upon the tooth crown contour point cloud dataset, the CloudCompare v2.10 point cloud processing software (CloudCompare Team, Bordeaux, France) was employed to extract feature point clouds corresponding to the gingival margin line and its three subregions, namely, the left posterior tooth area, anterior tooth area, and right posterior tooth area. The segmentation of the three subregions of the gingival margin line is shown in Figure 6. Figure 7 displays the mandibular and maxillary gingival margin details from three cases along with their corresponding division into the three subregions. We define these features as local features.

### 2.4. Feature Extraction Stage

In the feature extraction stage, two modules are included: point cloud downsampling and feature description based on machine learning-related algorithms. These modules extract feature information from the samples to assist in the subsequent identification stage.

#### 2.4.1. Point Cloud Downsampling

To effectively promote the efficiency of feature extraction while minimizing feature loss, in this study we employ the voxel downsampling method. This method involves partitioning the input point cloud using a regular voxel grid. The downsampled point for each voxel block is determined by calculating the center of gravity $G_{d}$ of all points within the block. This approach aims to retain the key characteristics of the original point cloud. The expression of the center of gravity $G_{d}$ is expressed as follows:

(1) $$G_{d}=\frac{\sum_{j=1}^{N_{d}}\left(x_{j},y_{j},z_{j}\right)}{N_{d}},\;d\in \left[1,2,\ldots ,m\right]$$

where $G_{d}$ represents the center of gravity of the d−th voxel block, $N_{d}$ denotes the number of points contained within this block, and the parameter *m* signifies the total number of voxel blocks encompassing points.

**Figure 8:**
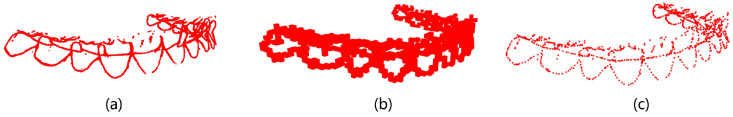
Schematic of mandibular model voxelization and downsampling: (**a**) initial point cloud with 14,841 points, (**b**) point cloud after voxelization with 1067 voxels, and (**c**) point cloud after downsampling with 1067 points.

In Figure 8, (a) showcases the point cloud before voxelization, (b) depicts the voxelized point cloud with a voxel grid resolution of 1, and (c) illustrates the point cloud after voxel downsampling. Noticeably, the downsampling process results in a reduction in the input point cloud data of about 92.8%.

#### 2.4.2. Geometric and Structural Feature Description of Dental Point Clouds

**Figure 9:**
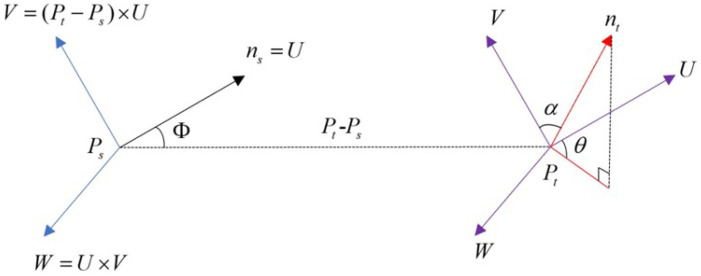
Local coordinate system; the meanings of the letters are explained in the text.

After downsampling the samples, we employed the FPFH [18] descriptor to characterize the features of the downsampled data. Based on the point feature histogram (PFH) [19] feature descriptor, FPFH simplifies the computational complexity and dimensions of the PFH feature descriptor. The processing steps [20,21] of FPFH feature description are as follows:

(1) A local coordinate system is established utilizing the query point $P_{s}$, its neighboring point $P_{t}$, and the normal vector $n_{s}$. As shown in Figure 9, this system positions $P_{s}$ as the origin and defines *U*, *V*, and *W* as the coordinate axes. This setup facilitates the description of spatial features between the two points.

In Figure 9, the expressions for the axes *U*, *V*, and *W* are as shown below.

(2) $$U=n_{s}$$

(3) $$V=U\times \frac{\left(P_{t}-P_{s}\right)}{\|P_{t}-P_{s}{\|}^{2}}$$

(4) W=U×V

In Equation (3), $\left|\right|P_{t}-P_{s}{\left|\right|}^{2}$ represents the square of the Euclidean distance between the query point $P_{s}$ and its neighborhood point $P_{t}$, while × means the cross-product.

Using these three axes, the three parameters α, ϕ, and θ in Figure 9 can be calculated, thereby establishing the spatial characterization of these two points. The calculation formulae are as follows:

(5) $$\alpha =V\cdot n_{t}$$

(6) $$\phi =U\cdot \frac{P_{t}-P_{s}}{\|P_{t}-P_{s}{\|}^{2}}$$

(7) $$\theta =\arctan(W\cdot n_{t},U\cdot n_{t})$$

where $n_{t}$ in Equations (5) and (7) represents the normal vector of the neighborhood point $P_{t}$.

(2) With the query point $P_{s}$ as the center point and a specified neighborhood radius *k*, we use the K-nearest neighbors algorithm to collect all points in the neighborhood. We then calculate the spatial feature descriptions of the query point $P_{s}$ and remaining points ($P_{k1}∼P_{kn}$) in its neighborhood to obtain *n* feature description triple parameters (α,ϕ,θ).

(3) The value ranges of the three parameters are partitioned into 11 sub-intervals, yielding a total of 33 intervals. Subsequently, the distribution of the triple parameters in each dimension is individually tallied and the outcomes are aggregated into histogram format for statistical analysis. These results are referred to as simple point feature histograms (SPFHs).

(4) Utilizing all neighborhood points except the query point $P_{s}$ from step (2) as new centroids, an SPFH is computed for each point. These results are then aggregated to form the FPFH feature descriptor of the query point $P_{s}$ through weighted averaging, as expressed below.

(8) $$FPFH\left(P_{s}\right)=SPFH\left(P_{s}\right)+\frac{1}{n}\sum_{i=1}^{n}\frac{1}{\omega_{i}}\cdot SPFH\left(P_{ki}\right)$$

In Equation (8), *n* represents the total count of neighborhood points acquired by query point $P_{s}$ with *k* as the neighborhood radius, while $\omega_{i}$ signifies the Euclidean distance from the *i*-th neighborhood point $P_{ki}$ to the query point $P_{s}$.

**Figure 10:**
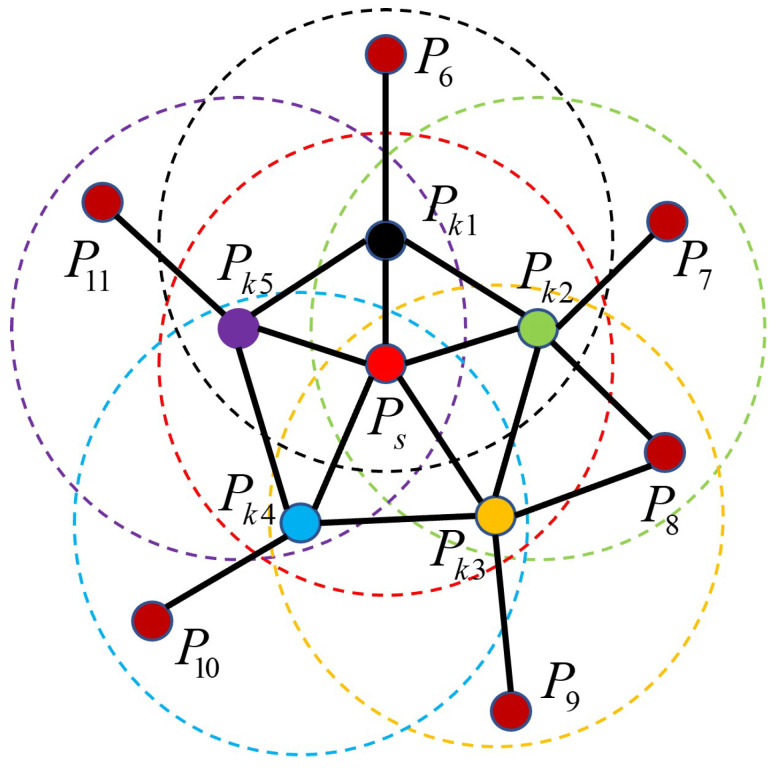
Schematic of FPFH feature neighborhood construction: $P_{s}$ denotes the query point (highlighted in red), with its surrounding neighborhood represented by a red dotted circle; points $P_{k1}$ to $P_{k5}$ within this circle serve as neighbors to $P_{s}$. Similar conventions apply to the other dots and dotted circles. Point names are solely for differentiation.

The range of influence for computing the FPFH feature descriptor is illustrated in Figure 10.

**Figure 11:**
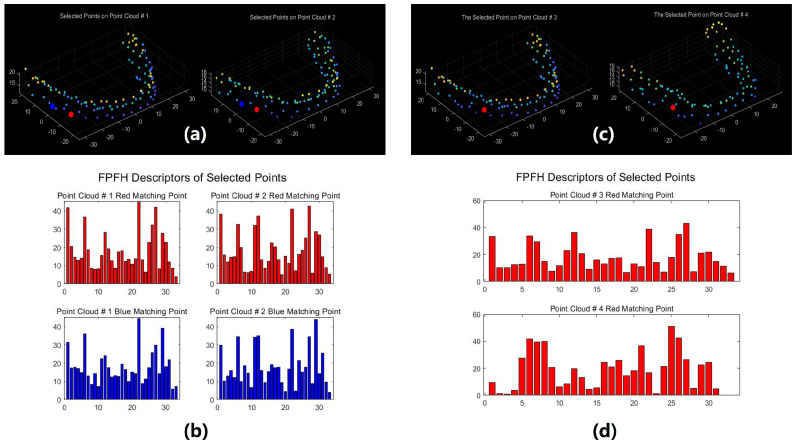
FPFH feature comparison: (**a**) two matching point pairs from point clouds with identical identity information; (**b**) FPFH feature for two matching point pairs; (**c**) non-matching points from point clouds with differing identities; (**d**) FPFH feature for non-matching points.

Figure 11 illustrates a comparison of FPFH features between matching pairs from two point clouds sharing identical identity information and a non-matching pair from two distinct point clouds. In Figure 11a, two matching point pairs, distinguished by color consistency (each color denoting a matching pair), are depicted among the four points; Figure 11b presents the FPFH histograms corresponding to the red and blue pairs, Figure 11c showcases a pair of points originating from point clouds with differing identities, and Figure 11d showcases the FPFH histogram associated with the points in Figure 11c. The analysis of Figure 11 reveals that data points within the same point cloud possessing similar structural features demonstrate more consistent FPFH features. Conversely, non-matching points exhibit different FPFH features even if they occupy the same position in the model, indicating that their structural features are different.

### 2.5. Registration-Based Identification Stage

In the registration-based identification stage, our method employs a coarse-to-fine registration process based on machine learning-related algorithms to align the query model with each gallery set model. The identity of the query model is then determined based on the registration results.

#### 2.5.1. Coarse Registration of Dental Point Clouds

Direct registration of two dental point clouds may be impossible due to differences in acquisition equipment or variations in dental morphology caused by factors such as age or disease. To facilitate the alignment of these models, an initial transformation matrix is needed to bring them into the same coordinate system. The process of obtaining this initial transformation matrix is called coarse registration. This process relies on extracting geometric and structural features rather than utilizing raw 3D coordinates, which aims to enhance the robustness of the transformation. After characterizing the downsampled source and target point clouds using the FPFH feature descriptor, the two point clouds can be roughly aligned using the SAC-IA algorithm [18,22]. The steps for applying the SAC-IA algorithm for point cloud alignment are as follows:

(1) Take *t* random data points in the source point cloud, and use the KD tree [23,24] algorithm to find points with similar FPFH features corresponding to these *t* points in the target point cloud to form *t* groups of point pairs.

(2) Use these *t* sets of point pairs to obtain the transformation matrix for transformation of the source point cloud to the target point cloud.

(3) Utilize the transformation matrix obtained in step (2) to transform the point clouds prior to voxelization downsampling.

(4) Evaluation of the transformation result derived from the transformation matrix involves assessing the metrics of fitness and root mean square error (RMSE). The fitness is calculated by dividing the number of matched pairs between the transformed point cloud (from step 3) and the target point cloud by the total points in the transformed point cloud, which serves to gauge the alignment degree. The RMSE indicates the alignment accuracy, and is computed as the root mean square error of the Euclidean distances between all corresponding point pairs from the transformed and target point clouds.

(5) The four steps outlined above are iteratively repeated for a predetermined number of iterations in order to acquire the transformation matrix which exhibits the highest fitness value and lowest RMSE throughout the iterations.

The SAC-IA algorithm facilitates swift acquisition of the transformation matrix for initial point cloud alignment, establishing a robust basis for subsequent fine alignment.

#### 2.5.2. Fine Registration of Dental Point Clouds

The point clouds aligned via the SAC-IA algorithm exhibit rough correspondence; however, the alignment accuracy remains insufficient for human identification. To enhance the alignment accuracy, in this study we utilized the ICP algorithm for fine registration based on the initial alignment achieved by the SAC-IA. The ICP algorithm, initially introduced by Besl and McKay [25], was employed for point cloud alignment in the following manner:

(1) The transformation matrix obtained from the SAC-IA serves as the initial transformation matrix for the ICP algorithm. This matrix is applied to the source point cloud in order to yield the initial source point cloud $P_{Source}$ for registration.

(2) A direction vector threshold is employed to search for the nearest point from the target point cloud $Q_{T}$ for each point in the source point cloud $P_{Source}$, forming a set of nearest neighbor pairs $\left(p_{h},q_{h}\right)$ comprising *N* pairs.

(3) The rotation matrix $R_{N}$ and translation matrix $T_{N}$ between the source point cloud $P_{Source}$ and the target point cloud $Q_{T}$ are calculated based on this set of nearest neighbor pairs, aiming to minimize the mean square error as defined in Equation (9).

(9) $$E\left(R_{N},T_{N}\right)=\frac{1}{N}\sum_{h=1}^{N}{∥q_{h}-\left(R_{N}p_{h}+T_{N}\right)∥}^{2}$$

(4) The rotation matrix $R_{N}$ and translation matrix $T_{N}$ obtained in the preceding step are utilized to construct a transformation matrix, which is then applied to the source point cloud $P_{Source}$ to generate a new source point cloud $P_{Source}^{\prime }$. Subsequently, the distance error between the updated source point cloud $P_{Source}^{\prime }$ and target point cloud $Q_{T}$ is calculated. If the number of iterations exceeds the predefined threshold or the distance error falls below a specified threshold, then the iteration terminates and the final transformation matrix comprising $R_{N}$ and $T_{N}$ is output to yield the ultimate registration outcome. Otherwise, the initial source point cloud is updated to $P_{Source}^{\prime }$ and steps (2)–(4) are repeated.

#### 2.5.3. Identification Method

The query model is registered against each model in the gallery set one-by-one and the corresponding fitness and RMSE values are output as the final registration results. The identity information of the gallery model with the smallest RMSE value when registered with the query model is used as the identification information of the query model.

## 3. Results and Discussion

### 3.1. Experimental Design

Our experiment comprised three components. First, we leveraged the holistic feature identification samples to verify the effectiveness of the proposed framework for human identification. Second, we explored the use of local feature identification samples for human identification to test the stability of the framework’s performance under conditions of partial tooth loss. Finally, we set up comparative experiments to demonstrate the superiority of the proposed framework.

#### 3.1.1. Experimental Design with Holistic Feature Identification Samples

The experimental procedure for using the holistic feature identification samples for human identification is as follows:

(1) Establish a tooth identity gallery set and a query set. The holistic feature identification samples corresponding to the initial scan models were stored in the gallery set for record-keeping purposes. One year later, the holistic feature identification samples extracted from the models of the second scan were assigned to the query set for evaluation of the proposed identification method.

(2) Identification experiments were conducted in which each query model was registered with all gallery models one-by-one using our proposed framework for human identification. The identity of the gallery sample with the lowest RMSE value was assigned to the query sample.

(3) The results of the identification process from step (2) were recorded and used to visualize the performance of the proposed framework.

#### 3.1.2. Experimental Design with Local Feature Identification Samples

The experimental procedure for identification utilizing the local feature identification samples is outlined as follows:

(1) Gallery and query sets were established based on the extraction method described in Section 2.3 and the tooth crown contour point cloud data set in experiment 3.1.1; corresponding gallery sets and query sets were created for these four features.

(2) Identification experiments were conducted separately using four types of local feature identification samples.

(3) Comparative analysis of identification performance was conducted. We analyzed and compared the results obtained from experiment 3.1.1 with the outcomes from this experiment while focusing on the performance variation when different features were used for identification.

#### 3.1.3. Setup of the Comparative Experiments

To further prove the superiority of our proposed framework in human identification tasks, we designed a series of comparative experiments. The experiments consisted of two parts:

(1) Different registration methods used in the registration-based identification stage were compared in order to highlight the advantages of our coarse-to-fine registration approach.

(2) The identification results of our method were compared with those of other existing methods to further demonstrate the superiority of our method in human identification.

### 3.2. Experimental Environment

All experiments in this paper were conducted using the Python 3.7 interpreter in the Pycharm integrated development environment. The computer used for the experiments was equipped with an Intel Core i5-11260H CPU, Windows 11 operating system, and NVIDIA GeForce RTX 3050Ti GPU.

### 3.3. Experimental Results with Holistic Feature Identification Samples

**Figure 12:**
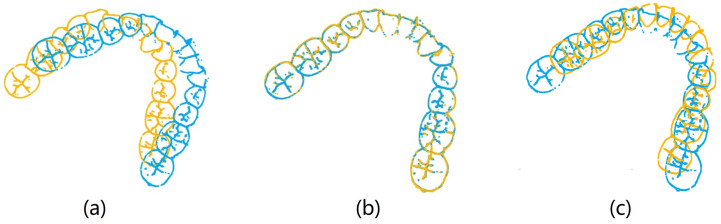
Visualization results of registration using holistic feature identification samples: (**a**) original holistic feature identification samples, (**b**) genuine match result, and (**c**) imposter match result.

The holistic feature identification samples were derived from the 320 primary and 152 secondary scanned models and the extracted results were cataloged into the gallery and query sets. First, we present and evaluate the experimental results through a qualitative analysis. We visualize the registration results of two holistic feature identification samples from the same individual (referred to as a genuine match) and the registration result of holistic feature identification samples from distinct individuals (referred to as an imposter match), as shown in Figure 12.

From the visualization results in Figure 12, it can be observed that two samples belonging to a genuine match align well after registration, while two samples belonging to an imposter match show significant misalignment after registration. The results demonstrate that our proposed framework effectively aligns two samples from the same individual during the registration-based identification stage using the coarse-to-fine registration method. Proper alignment enables the framework to accurately determine the identity of the query model.

**Figure 13:**
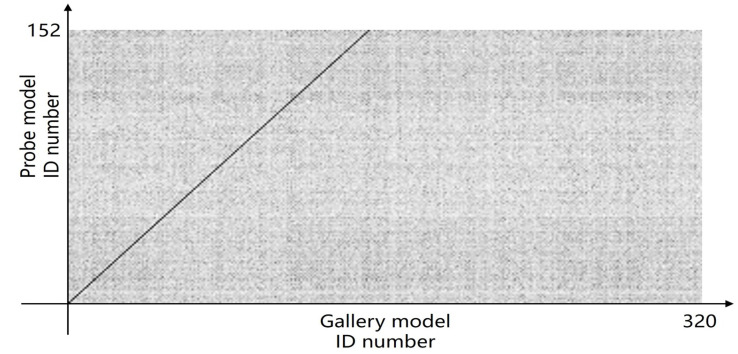
RMSE matrix pixmap of registration results.

**Figure 14:**
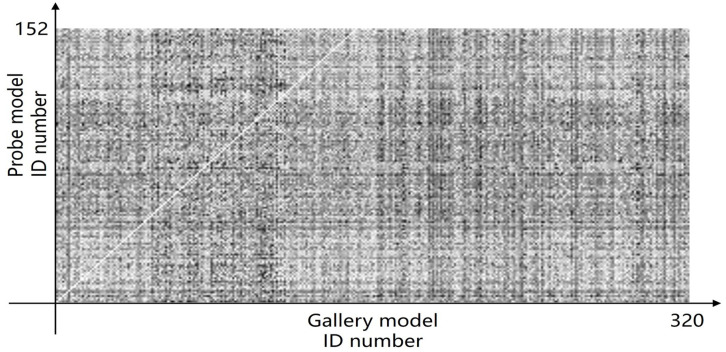
Fitness matrix pixmap of registration results.

Furthermore, we present and evaluate the experimental results through quantitative analysis. The RMSE and fitness values obtained from registering each query model with all models in the gallery set are organized into matrices. The subdiagonal elements of the RMSE matrix contain the registration values of two models with identical identity information, as does the fitness matrix. Notably, the subdiagonal elements of the RMSE matrix exhibit minimal values compared to other elements in the corresponding row, showing significant differences. Statistically, the RMSE values range from 0 to 1.5; however, the subdiagonal elements of the fitness matrix do not demonstrate significant deviation from the corresponding row elements. Across the fitness matrix, the values range from 0 to 1. Figure 13 and Figure 14 respectively illustrate the visualization of the RMSE and fitness matrices obtained by scaling the values of the matrix elements to the range of 0 to 255.

Figure 13 illustrates that the subdiagonal pixel values are minimal, indicating that the RMSE values of the genuine matches are significantly lower than the RMSE values of the imposter matches for all query samples. Conversely, Figure 14 indicates that for certain query samples there are insignificant disparities in the fitness values between genuine and imposter matches.

In Table 1, the first column presents statistical information regarding the RMSE and fitness. Analysis of the distribution of RMSE and fitness values for genuine matches (152 pairs) and impostor matches (48,488 pairs, with green indicating genuine matches and blue representing impostor matches) reveals that RMSE is a more reliable metric for human identification. The table also illustrates that our proposed method utilizing holistic feature identification samples for human identification achieves a Rank-1 recognition rate of 100%.

### 3.4. Experimental Results with Local Feature Identification Samples

The local feature identification samples were derived from the 320 primary and 152 secondary scanned models, with a total of four gallery sets and four query sets being obtained. These sets included the gingival line gallery set, the left posterior region gallery set, the anterior tooth region gallery set, and the right posterior region gallery set along with their corresponding query sets. The performance of human identification based on these four sets of features is summarized in Table 1 (columns 2 to 5).

Table 1 indicates that when utilizing the left posterior region or right posterior region of the gingival margin line for identification, the corresponding Rank-1 recognition rate does not achieve 100%. This is attributed to the reduced number of features and point cloud data, which leads to insufficient data features for the coarse-to-fine registration algorithm, resulting in identification failure. Conversely, both the tooth crown contour and gingival margin line features offer ample distinctive features for identification, resulting in complete separation of the intra-class RMSE distribution from the inter-class RMSE distribution. However, as the number of feature points decreases, the intra-class RMSE distribution starts to overlap with the inter-class RMSE distribution. The number of feature points provided by the corresponding features decreases in the following order: tooth crown contour > gingival line > anterior area > left posterior area = right posterior area, leading to varying Rank-1 identification performance results.

Regarding the fitness factor across these five characteristics, the intra-class fitness distribution overlaps with the inter-class fitness distribution. The highest fitness value for genuine matches and impostor matches can reach 1, and a substantial portion of impostor match fitness values fall between 0.9 and 1. These statistical findings underscore the superiority of using RMSE values as an indicator for human identification.

Additionally, the average RMSE for genuine matches is notably lower than for impostor matches, and the average fitness for genuine matches is significantly higher than for impostor matches. However, as the number of feature points decreases, these gaps gradually diminish.

While the recognition rates using the left posterior tooth area data and right posterior tooth area data fall short of 100%, they still exceed 96.05%. This experiment underscores the effectiveness of our proposed framework for human identification using IOS data. Moreover, it demonstrates that our framework maintains robust identification performance even in cases of partial tooth loss. Thus, our framework can assist forensic personnel in human identification tasks.

### 3.5. Comparative Experiment

#### 3.5.1. Comparison of Registration Methods

**Figure 15:**
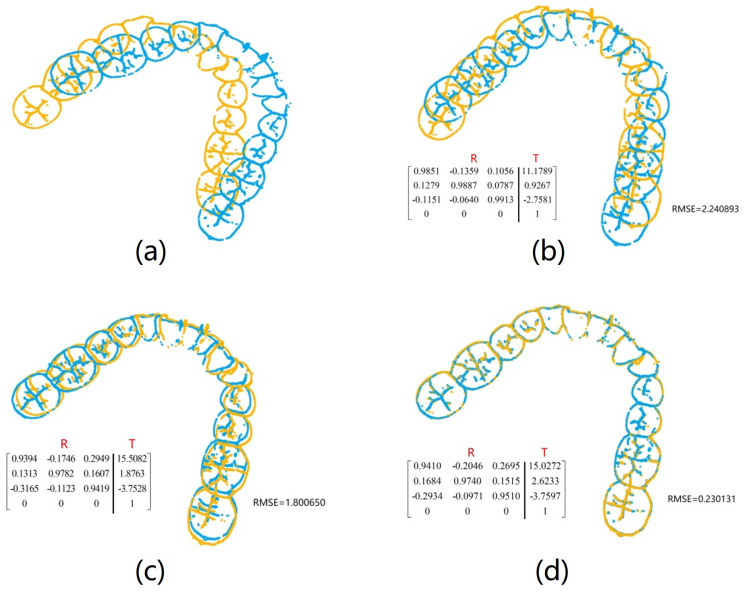
Comparison of SAC-IA registration, ICP registration, and coarse-to-fine registration: (**a**) original holistic feature identification samples, (**b**) registration result using the ICP method alone, (**c**) registration result using the SAC-IA method alone, and (**d**) registration result using the coarse-to-fine registration method alone. The transformation matrix between models (R: rotation matrix, T: translation vector) is shown in lower left corner of (b–d). The RMSE values in (b–d) are derived from the corresponding registrations.

To validate our coarse-to-fine registration method’s enhancement of the proposed framework’s performance in human identification tasks, we selected two holistic feature identification samples from the same individual. We then separately applied the SAC-IA registration method, the ICP registration method, and our coarse-to-fine registration method to register the samples. The results are shown in Figure 15.

The outcomes from various registration methods reveal that when the original two samples of the same individual are not located in the same coordinate system, direct application of the ICP method results in considerable deviation between the two samples, as depicted in Figure 15b. Registration utilizing the SAC-IA method demonstrates improved outcomes, with substantial overlap between the two samples. However, minor discrepancies persist that make this insufficient for reliable identification support, as shown in Figure 15c. Following SAC-IA registration, the ICP method is employed for refined registration of point clouds, resulting in near-complete overlap of the two samples, as illustrated in Figure 15d. In terms of RMSE values, coarse-to-fine registration yields significantly lower values compared to SAC-IA or ICP registration alone, indicating superior registration performance. The results presented in Figure 15 comprise both visual registration and RMSE calculations, confirming the practicality and precision of employing coarse-to-fine registration for registering 3D dental point clouds.

#### 3.5.2. Comparison of Related Results

Both Reesu et al. [13] and Mou et al. [15] employed similar methodologies to superimpose IOS data for use in human identification, with both achieving a Rank-1 accuracy of 100%. We compared our results with [13,15] across two key aspects.

Table 2 compares the identification performance using different tooth feature samples and different dataset sizes. Regarding the tooth feature samples used in identification, the previous studies [13,15] utilized the entire dentition point cloud without performing feature extraction. In contrast, we focus on extracting features closely related to tooth structure for identification and study the identification task under conditions where tooth features are severely missing. Our experimental results show that utilizing tooth crown contour feature identification samples from IOS achieves 100% accuracy in human identification. When using the local feature identification samples, the accuracy rate exceeds 96.05%. Although the accuracy of human identification using the local feature identification samples has not yet reached 100%, it can be used as a supplementary method for identification when tooth crown contour features cannot be fully obtained due to uncontrollable events. Moreover, our dataset differs markedly in scale compared to those in [13,15].

Table 3 compares the efficiency and recognition rates of the different methods. The methodologies utilized by [13,15] were both essentially ICP-based 3D superposition techniques; thus, we conducted registration and identification experiments using the ICP method on dentition and tooth crown contour data and compared these results with our proposed method. From Table 3, it is evident that the Rank-1 recognition rate achieved when directly applying the ICP method to superimpose dentition data for human identification falls short of 100%. This further underscores the limitations of the methods employed in [13,15] when dealing with larger datasets. Moreover, the recognition rate results in the first and second columns indicate that the crucial identification features are retained in the extracted tooth crown contour features. Further tooth feature extraction reduces the point cloud data volume, significantly enhancing registration efficiency. Compared to the methods advocated by [13,15], our proposed method demonstrates superior recognition rates and processing speed.

Based on the comparative experimental results presented in this section, it is evident that the proposed method outperforms previous approaches in terms of both efficiency and recognition accuracy for human identification. Moreover, our study evaluates the performance of the proposed method under conditions of partial tooth loss, which has not been covered by prior research. Our test results confirm that the proposed method maintains high identification performance even in cases of partial tooth loss.

However, due to the lack of publicly available IOS datasets on the internet, the sample size and geographical diversity of the data used in our study are limited. In future research, we will further expand the sample size and enhance its geographical diversity.

In order to assess the generalizability of the research findings, we conducted an analysis from two perspectives. First, from the viewpoint of technical implementation complexity, the framework employed in this study does not involve model training, instead focusing on optimizing machine learning-related algorithms to enhance their applicability in human identification based on 3D dental point clouds. Consequently, the proposed framework exhibits lower implementation complexity and higher generalizability. Second, intraoral scanning technology is widely used around the world due to its ease of operation and ability to collect accurate data. The proposed framework can directly utilize IOS data collected from different regions and different populations for human identification. From this perspective, our framework demonstrates high generalizability.

## 4. Conclusions

Presently, the majority of dental biometrics-based human identification works rely on panoramic dental X-ray images or digitized dental plaster models as the primary samples for identification processing.

However, with the continuous advancement of digital technology, intraoral scanning technology is expected to become a common method for sample collection in the field of dental biometrics-based human identification.

This study proposes a three-stage human identification framework based on IOS data. In the first stage, the curvature principle is used to extract discriminative holistic feature identification samples (tooth crown contours) from IOS data while filtering out most of the irrelevant points and retaining the key features. Additionally, four local feature identification samples are constructed to assess the framework’s effectiveness in cases of partial tooth loss. The second stage involves extracting geometric and structural features from the samples to assist in the subsequent registration-based identification stage. Finally, the third stage implements a coarse-to-fine registration process to complete the human identification task based on dental point cloud registration.

In general, using the framework proposed in this paper, the Rank-1 recognition rate of human identification using tooth crown contour features is 100%, while the Rank-1 recognition rates of human identification using other local features are all above 96.05%. These results demonstrate that the proposed method can effectively utilize IOS data for human identification while maintaining excellent performance even in cases of partial tooth loss. In the future, researchers can build upon the three-stage framework proposed in this paper by incorporating additional machine learning-based models to enhance the feature extraction and registration of 3D dental point clouds, thereby further improving the efficiency of human identification. In addition, the framework proposed in this paper can be used as a baseline to evaluate the performance of related improvement methods.

## Figures and Tables

**Table 1:** Performance comparison of identification using five different dental features.

Type	1	2	3	4	5
Feature name	the tooth crown contour	the gingival margin line	the left posterior tooth area	the anterior tooth area	the right posterior tooth area
The number of query models	152	152	152	152	152
The number of gallery models	320	320	320	320	320
Rank-1 recognition rate	100%	100%	96.05%	100%	98.03%
Genuine match RMSE maximum (mm)	0.434	0.381	0.973	0.359	0.907
Genuine match RMSE minimum (mm)	0.109	0.082	0.058	0.067	0.068
Impostor match RMSE maximum (mm)	1.368	1.383	1.316	1.339	1.293
Impostor match RMSE minimum (mm)	0.655	0.485	0.205	0.227	0.215
Genuine match RMSE average (mm)	0.198	0.182	0.175	0.144	0.156
Impostor match RMSE average (mm)	1.140	1.083	0.844	0.898	0.841
Distribution of RMSE results for genuine matches and impostor matches	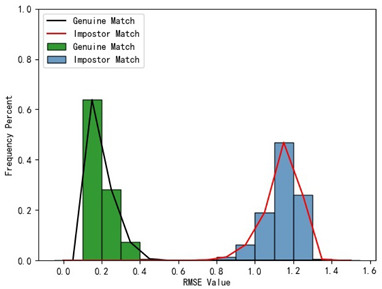	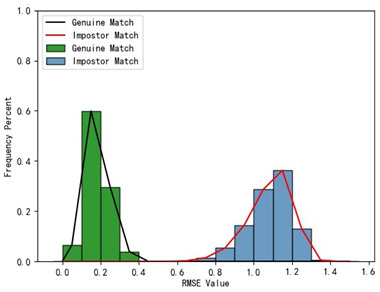	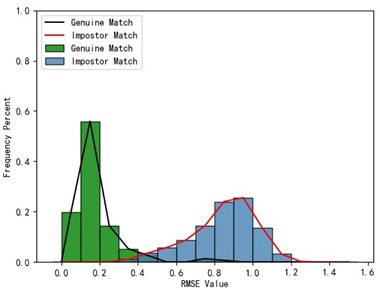	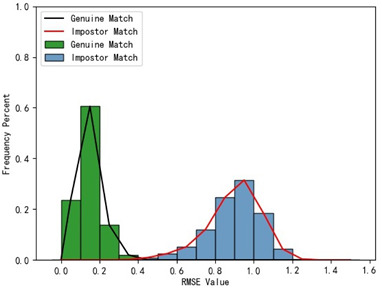	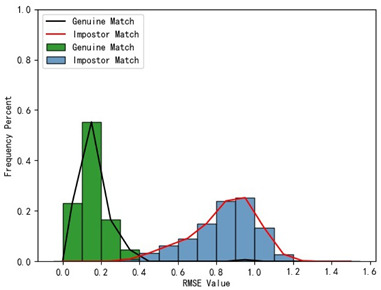
Genuine match Fitness maximum	1	1	1	1	1
Genuine match Fitness minimum	0.918	0.928	0.778	0.965	0.862
Impostor match Fitness maximum	0.999	1	1	1	1
Impostor match Fitness minimum	0.057	0.062	0.079	0.073	0.087
Genuine match Fitness average	0.997	0.997	0.994	0.999	0.998
Impostor match Fitness average	0.660	0.620	0.800	0.832	0.805
Distribution of Fitness results for genuine matches and impostor matches	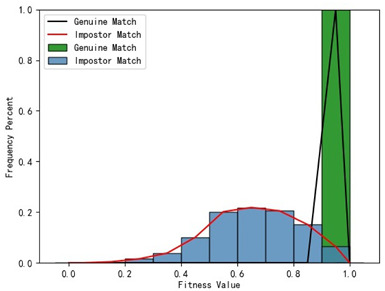	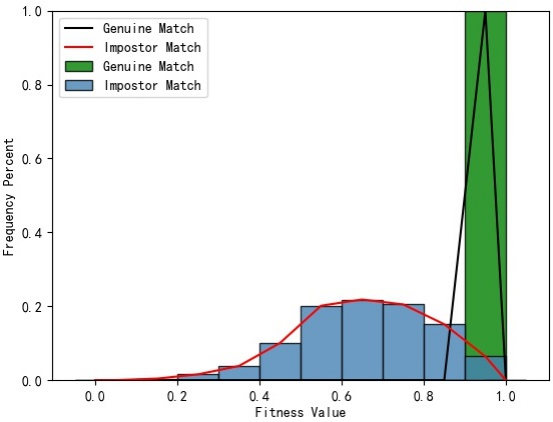	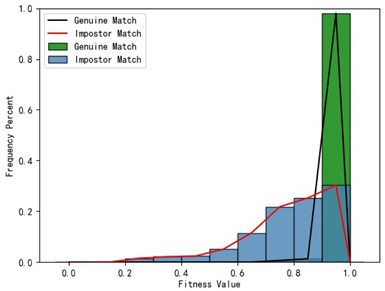	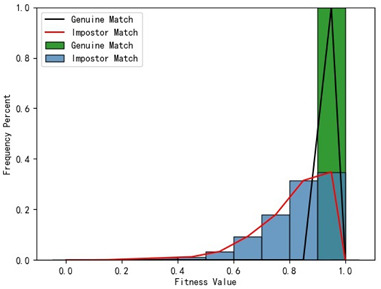	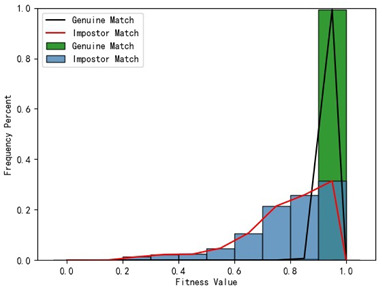

**Table 2:** Comparison of our experiments with those from Reesu et al. [13] and Mou et al. [15] on tooth feature, identification effect, and dataset size.

	Reesu	Mou	Ours (F1)	Ours (F2)	Ours (F3)	Ours (F4)	Ours (F5)
Gallery model number	60 (IOS)	100 (3D model transferred from plaster casts)	320 (IOS)	320 (IOS)	320 (IOS)	320 (IOS)	320 (IOS)
Query model number	60 (IOS)	28 (IOS)	152 (IOS)	152 (IOS)	152 (IOS)	152 (IOS)	152 (IOS)
Rank-1 recognition rate	100%	100%	100%	100%	96.05%	100%	98.03%

F1 represents the tooth crown contour, F2 represents the gingival margin line, F3 represents the left posterior tooth area, F4 represents the anterior tooth area, and F5 represents the right posterior tooth area.

**Table 3:** Comparison of our method with those of Reesu et al. [13] and Mou et al. [15] in terms of efficiency and Rank-1 recognition rate.

	The Entire Dentition + ICP	The Tooth Crown Contour + ICP	The Tooth Crown Contour + Coarse-to-Fine Registration (Ours)
Number of point clouds	85,680	15,838	15,838
Rank-1 recognition rate	96.71%	96.71%	100%
Registration time	0.896 s	0.085 s	0.142 s

## Data Availability

The datasets analyzed during the current study are not publicly available due to confidentiality, but are available from the corresponding author on reasonable request.
